# The Global Challenge of *Campylobacter*: Antimicrobial Resistance and Emerging Intervention Strategies

**DOI:** 10.3390/tropicalmed10010025

**Published:** 2025-01-16

**Authors:** Zubeiru Bukari, Toyin Emmanuel, Jude Woodward, Richard Ferguson, Martha Ezughara, Nikhil Darga, Bruno Silvester Lopes

**Affiliations:** 1 School of Health and Life Sciences, Teesside University, Middlesbrough TS1 3BX, UK; 2 National Horizons Centre, Teesside University, Darlington DL1 1HG, UK

**Keywords:** *Campylobacter*, antimicrobial resistance, fluoroquinolones, macrolides, tetracyclines, aminoglycosides, transmission, interventions

## Abstract

Antimicrobial resistance (AMR) in *Campylobacter* species, particularly *C. jejuni* and *C. coli*, poses a significant public health threat. These bacteria, which are commonly found in livestock, poultry, companion animals, and wildlife, are the leading causes of foodborne illnesses, often transmitted through contaminated poultry. Extensive exposure to antibiotics in human and veterinary medicine creates selection pressure, driving resistance through mechanisms such as point mutations, horizontal gene transfer, and efflux pumps. Resistance to fluoroquinolones, macrolides, and tetracyclines complicates treatment and increases the risk of severe infections. Drug-resistant *Campylobacter* is transmitted to humans via contaminated food, water, and direct contact with animals, highlighting its zoonotic potential. Addressing this challenge requires effective interventions. Pre-harvest strategies like biosecurity and immune-based methods reduce bacterial loads on farms, while post-harvest measures, including carcass decontamination and freezing, limit contamination. Emerging approaches, such as bacteriocins and natural antimicrobials, offer chemical-free alternatives. Integrated, multidisciplinary interventions across the food chain are essential to mitigate AMR transmission and enhance food safety. Sustainable agricultural practices, antimicrobial stewardship, and innovative solutions are critical to curbing *Campylobacter* resistance and protecting global public health. Our review examines the dynamics of antimicrobial resistance in *Campylobacter* and presents current strategies to mitigate *Campylobacter*-related AMR, offering valuable insights for antimicrobial control in the poultry industry.

## 1. Introduction

Antimicrobial resistance (AMR) refers to the process by which bacteria, fungi, parasites, and viruses evolve to the point where they cannot be treated with antimicrobial drugs like antibiotics [1]. It is a major global health concern posing serious threats to the effectiveness of antibiotics and other antimicrobial medicines, essential for treating infections in humans and animals [2]. With around 48 million cases recorded annually among children under five years globally, *Campylobacter* spp. is a major cause of diarrhoea in children worldwide [3]. Chickens are considered a natural reservoir of *Campylobacter* spp., particularly *Campylobacter jejuni*, because their intestinal system provides an ideal biological habitat for the survival and growth of these bacteria owing to their high body temperature. Though they are mostly asymptomatic after colonization, chickens usually become colonized by the time they are two to three weeks old [4].

Among the pathogens associated with AMR, *Campylobacter* species, especially *C. jejuni* and *C. coli,* are the major causes of gastrointestinal infections in humans, with children being the most susceptible [5]. They are gram-negative bacteria, spiral or curved in shape, belonging to the phylum Proteobacteria [6]. *Campylobacter* is the most common cause of foodborne illnesses worldwide and causes an illness called campylobacteriosis in humans [7]. A recent study conducted in the UK showed that *Campylobacter* is a common cause of gastroenteritis, affecting as many as 630,000 people and leading to 80,000 GP consultations annually [5,8]. These infections are mostly zoonotic, with poultry being the main reservoir [9]. Over 200 people die from its effects every year in the UK, leading to an estimated economic loss of around GBP 1 billion each year [10]. Humans contract infections primarily by consuming contaminated animal-based foods, particularly undercooked poultry meat, unpasteurised milk, and dairy products [11]. While infections typically result in less severe diarrhoea, they can also cause severe chronic or systemic infections in young children, the elderly, and immunocompromised individuals. *Campylobacter* infections are linked to various complications, including the non-paralytic form of Guillain–Barré Syndrome (GBS), Miller–Fisher syndrome, and reactive arthritis. Severe outcomes can arise from the infection spreading beyond the gastrointestinal tract, resulting in conditions such as cholecystitis, pancreatitis, peritonitis, or significant gastrointestinal bleeding. Although infrequent, extra-intestinal manifestations have been reported, including meningitis, endocarditis, septic arthritis, osteomyelitis, and neonatal sepsis [12].

In extreme cases in immunocompromised patients, treatment with antibiotics such as fluoroquinolone, macrolides, or aminoglycoside may be required [13]. Concerns over the sustainability of present treatment options have been raised by the widespread use of antibiotics in animal farming, which has led to the selection and dissemination of resistant strains [14]. Antibiotic resistance in *Campylobacter* is becoming more and more common because of the bacterial ability to develop several resistance mechanisms, which affect their efficacy, raising major health concerns [15]. As a result, effective intervention strategies are crucial to reducing threats to public health, especially considering the dual concerns of AMR and the worldwide prevalence of *Campylobacter* infections [13]. These intervention strategies, including biosecurity measures and carcass decontamination, have been put in place to reduce the burden of these bacteria in poultry [4]. This review looks at the dynamics of AMR in *Campylobacter*, highlighting the current intervention strategies and providing a comprehensive understanding of approaches to mitigate *Campylobacter*-related AMR. It contributes to the global discourse on antimicrobial stewardship and interventions (physical and natural chemical methods) across the poultry sector.

## 2. Development and Transmission of Antimicrobial Resistance in *Campylobacter*

### 2.1. Mechanisms of Resistance and Bacterial Evolution

The development of antimicrobial resistance (AMR) in *Campylobacter* reflects the ongoing evolutionary adaptation of bacteria to environmental and therapeutic pressures [16]. Resistance mechanisms in *Campylobacter*, including point mutations and gene acquisition, enable these bacteria to withstand commonly used antibiotics, posing significant public health challenges [17]. For instance, single-point mutations in the DNA gyrase (*gyr*A) gene, which encodes a type II topoisomerase essential for DNA replication, transcription, and repair, confer fluoroquinolone resistance (FQ-R) by reducing drug binding affinity [18,19]. Similarly, mutations in the 23S rRNA gene lead to macrolide resistance by decreasing the binding of antibiotics to their ribosomal targets [13]. These genetic alterations are key drivers of AMR in *C. jejuni* and *C. coli*, the most clinically significant species.

Horizontal gene transfer (HGT) plays a pivotal role in disseminating resistance genes among *Campylobacter* populations. Mechanisms such as conjugation, transformation, and transduction facilitate the acquisition of resistance determinants from other bacterial species, further accelerating the spread of AMR [11]. For example, the *cme*ABC multidrug efflux pump, encoded on transferable genetic elements, is a well-characterized system in *Campylobacter* that contributes to resistance against fluoroquinolones, macrolides, and tetracyclines [20]. Moreover, plasmid-mediated resistance genes, such as *tet*(O) for tetracycline resistance, underscore the role of HGT in the rapid adaptation of *Campylobacter* to antimicrobial pressure [21].

Vertical gene transfer (VGT), driven by the natural selection of advantageous mutations, also contributes to the persistence and propagation of resistant strains. This process is particularly significant in environments with high antibiotic usage, such as poultry farms, where *Campylobacter* populations are under constant selective pressure [22]. The co-occurrence of HGT and VGT creates a dynamic interplay, fostering genetic diversity and enabling *Campylobacter* to adapt to diverse environmental and host conditions.

Understanding the mechanisms of resistance in *Campylobacter* is crucial for developing effective mitigation strategies. Targeting key resistance pathways, monitoring the spread of resistance genes through HGT, and implementing antimicrobial stewardship programs are critical steps in combating the rise of AMR in this pathogen. Research on the adaptation of *Campylobacter* to commonly used antibiotics remains essential, as it provides insights into the evolutionary dynamics underlying resistance development. Recent studies have highlighted the global distribution of resistant *Campylobacter* strains, emphasizing the urgent need for a coordinated approach to manage AMR in both clinical and agricultural settings [4,15].

### 2.2. Selective Pressure, Fitness Cost, and Adaptive Compensations

Selective pressures, particularly the widespread use of antibiotics in clinical and agricultural settings, amplify the evolutionary success of resistant *Campylobacter* strains. For instance, fluoroquinolones, macrolides, tetracyclines, and aminoglycosides exert significant selective pressure, favouring the survival of resistant strains [23,24,25]. This constant pressure accelerates the evolutionary process, allowing resistance traits to become fixed within bacterial populations.

While resistance mechanisms often impose metabolic costs on bacteria, *Campylobacter* exhibits remarkable adaptability. Resistance-conferring mutations or acquired genes can initially reduce bacterial growth and competitiveness. However, the evolutionary process compensates for these fitness costs through secondary, compensatory mutations that restore or even enhance bacterial fitness [26,27]. This balance between fitness cost and compensatory evolution underscores the resilience of *Campylobacter* in the face of antibiotic interventions.

### 2.3. Transmission Pathways

The primary cause of *Campylobacter* infection in humans is the consumption of undercooked or contaminated food and contaminated water from taps, ponds, and boreholes, with people residing in rural areas being at a higher risk from contaminated private water supplies [28,29,30]. It can also be transmitted through contact with infected animals and faecal–oral or via fomites. Resistant strains can enter the food chain either through contaminated meat or cross-contamination during food preparation leading to infection [31].

**Figure 1:**
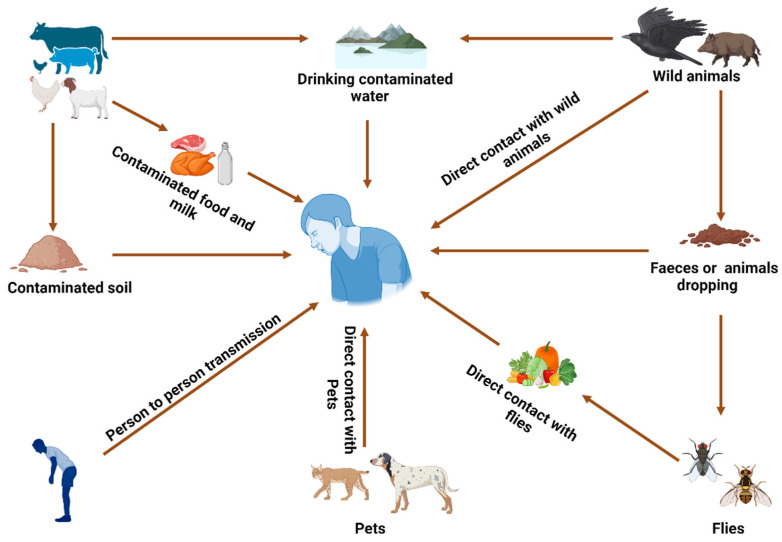
Transmission pathways in *Campylobacter*.

Again, biofilms in processing facilities have also been said to contribute to ways that harbour and protect resistant *C. jejuni*. Other contributing factors involve direct contact with wildlife and livestock, especially poultry and cattle, which can transmit resistant *Campylobacter* [32]. Though rare, transmission of *Campylobacter* infections can occur in healthcare or household settings where hygiene practices are very poor and inadequate (Figure 1).

## 3. Mechanisms of Antimicrobial Resistance

### 3.1. Resistance to β-Lactams

Beta-lactams comprise a class of antimicrobials including penicillin, cephalosporins, carbapenems, and monobactams. β-lactams inhibit bacterial cell wall synthesis via binding penicillin-binding proteins and interfering with the peptide cross-linking required in the biosynthesis of peptidoglycan [33]. In most cases, *Campylobacter* spp. is resistant to most β-lactams bar specific carbapenems [34,35]. In *Campylobacter*, there are three known mechanisms of β-lactam resistance: multidrug efflux pumps, enzyme-mediated inactivation of the antimicrobial molecule, and reductions in drug uptake into the cell [35,36]. β-lactamases are enzymes that hydrolyse the amide bond of the β-lactam ring within the structure of β-lactam antimicrobials, thus inactivating them [33].

The primary mechanism of β-lactam resistance in *Campylobacter* spp. is via the production of class D β-lactamases, also referred to as oxacillinases, which act to enzymatically hydrolyse β-lactam molecules. Class D β-lactamases are prevalent among many gram-negative microbes such as *Pseudomonas aeruginosa* and *Enterobacteriaceae*. Class D comprises narrow-spectrum β-lactamases, providing resistance to penicillin, ampicillin, amoxicillin, cloxacillin, and oxacillin (thus their name), with limited activity against first-generation cephalosporins [37]. There exist 14 families of class D beta-lactamases, many families comprising a single member, with most of the group comprising the 965 members of the OXA family [37]. Many gram-negatives have intrinsic OXA β-lactamases, with *Campylobacter jejuni* and *C. coli* having chromosomally located gene clusters for any one of the β-lactamases, such as *bla*_OXA-61-like_, *bla*_OXA-184-like_, *bla*_OXA-493-like_, and *bla*_OXA-576-like_ [37] (Table 1). Encoded by the *bla*_OXA-61_ gene, OXA-61 is the most studied and the most prevalent class D β-lactamase in *C. jejuni* conferring resistance to ampicillin, amoxicillin, and ticarcillin. Despite the β-lactamase activity, almost 50% of *bla*_OXA-61_ positive *C. jejuni* isolates can remain susceptible to ampicillin [38]. Levels of expression of the *bla*_OXA-61_ gene were suggested to impact the resistance phenotype. G → T transversion point mutation in the -57-promoter region upstream of *bla*_OXA-61_ were associated with increased OXA-61 expression and thus increased β-lactam resistance [39]. A deletion at position A69 of the *bla*_OXA-61_ promoter induces increased expression of *bla*_OXA-61_. When both the G57T mutation and A69 deletion were present, significant overexpression of *bla*_OXA-61_ was seen in *C. coli* [40]. While OXA-61-like and OXA-184-like are the most prevalent β-lactamases associated with *C. jejuni* and *C. coli*, other OXA family β-lactamases are present in different *Campylobacter* species and strains. OXA-493 and OXA-518 have been identified in isolates of *C. lari* conferring basal level of resistance to β-lactam antibiotics [41]. The *bla*_OXA-184_ has also been described in *C. jejuni* sequence type (ST) 51 isolates [42].

### 3.2. Resistance to Fluoroquinolones

Discovered in the 1960s, quinolones have since become important therapies in the treatment of community-acquired and severe nosocomial infections [64]. Quinolones pose several desirable characteristics, such as a broad spectrum of activity, limited incidence of severe side effects, high potency, and good bioavailability [64,65,66], and, as such, have been widely prescribed for a variety of infections [64,66].

Fluoroquinolones, such as ciprofloxacin, are among the primary treatments for *Campylobacter* infections in both humans and animals [67]. Quinolone and fluoroquinolone resistance among clinically important *Campylobacter* strains is increasing in prevalence globally, limiting the utility of currently used antimicrobials [67]. *Campylobacter* strains are commonly found to asymptomatically colonize the gastrointestinal tracts of livestock animals such as cattle, where they are regularly exposed to antimicrobials meant for the control and treatment of other pathogens [68]. In the case of cattle, particularly calves, metaphylactic treatments using fluoroquinolones, enrofloxacin, and danofloxacin are used where there is a high risk of developing bovine respiratory disease, such as during periods of stress, overcrowding, or after transportation [68]. As a foodborne pathogen, *Campylobacter* in livestock can spread to humans via the consumption of contaminated meats and other foods. In the UK and elsewhere, resistance to ciprofloxacin remains high among *C. jejuni* isolates from broiler farms (59% in the UK [69]). As of 2022, however, there was no use of fluoroquinolone antimicrobials on UK broiler farms following a steady decline in their use over previous years [69].

Quinolones act to inhibit bacterial DNA synthesis and cause cell death. Their mode of action comprises the inhibition of DNA gyrase and DNA topoisomerase IV, two enzymes responsible for chromosomal supercoiling during bacterial DNA synthesis [22,36,64]. thus interfering with DNA replication, repair, recombination, and transcription [22,66].

In general, the enzymes DNA gyrase and topoisomerase IV are each composed of two subunits (*gyr*A and *gyr*B as well as *par*C and *par*E, respectively) [70]. Quinolone resistance is often conferred via mutations in one or more of these subunits [70]. *Campylobacter* lacks the genes (*par*C and *par*E) that encode topoisomerase IV; thus, all fluoroquinolone resistance in *Campylobacter* is mediated by the mutations of DNA gyrase-encoding genes [52,71,72]. Generally, mutations in *gyr*B have not been found to confer antimicrobial resistance [72]. Point mutations in the quinolone resistance-determining region of *gyr*A are the primary resistance mechanism to fluoroquinolones [11,72], with a single point mutation in this region capable of significantly decreasing susceptibility [11,72,73]. Multiple different *gyr*A modifications, i.e., D90N, A70T, T86K, and T86I, have been reported to confer fluoroquinolone resistance [53,74,75] (Table 1). The C257T change in the *gyr*A gene, which leads to a T86I change in *gyr*A, is the most common modification observed in fluoroquinolone-resistant strains [11,74,76]. Along with *gyr*A mutations, the *cme*ABC multidrug efflux pump, found commonly among *Campylobacter*, can enhance fluoroquinolone resistance, and studies have demonstrated that its inactivation leads to increased fluoroquinolone susceptibility [20,73,77,78,79].

### 3.3. Resistance to Tetracyclines

Tetracyclines are a class of antimicrobials that prevent aminoacyl-tRNA binding to the ribosomal acceptor (A) site, thus inhibiting bacterial protein synthesis [80,81,82]. Extensive use over previous decades has resulted in widespread resistance among several bacterial groups [80,81]. Tetracycline resistance follows one of four mechanisms: RNA mutations, alterations to the chemical structure of tetracycline, efflux pumps, and ribosomal protection proteins [36,50,83].

In *Campylobacter*, resistance to tetracycline is associated with *tet*(O), a ribosomal protection protein (RPP), and the efflux pumps *cme*ABC and *cme*G [36,80,83,84]. *tet*(O) confers resistance via binding to the A site of a bacterial ribosome, which in turn facilitates a conformational change that disrupts the binding of tetracycline and ejects the antimicrobial molecule [50]. Originating from horizontal transfer from a gram-positive organism [85,86,87], the *tet*(O) gene is widely found among *C. jejuni* and *C. coli*, present either within the chromosomal DNA or conferred via a plasmid such as pCC31 or pTet [86,88,89]. Through recombination, two or more RPP-encoding genes can form functional chimeras termed mosaic genes [90]. Most tetracycline-resistant mosaic genes comprise recombinations of the RPP genes *tet*(O), *tet*(W), and *tet*(32), with others including segments of *tet*(M) and *tet*(S). In *Campylobacter*, the mosaic genes *tet*(O/32/O) and *tet*(O/M/O) have previously been reported in human isolates [51,90] (Table 1). Efflux pumps such as *cme*ABC and *cme*G provide intrinsic resistance to several antimicrobials, including tetracycline, with the inactivation of either pump leading to increased susceptibility [20,78,91]. *tet*(O) and *cme*ABC can act synergistically, leading to decreased tetracycline susceptibility [20,92].

### 3.4. Resistance to Macrolides

Macrolides comprise a class of antimicrobials that act upon the 50S ribosomal subunit to obstruct peptide translocation, thus inhibiting protein synthesis [93,94]. Resistance to macrolides follows one of three mechanisms: multidrug efflux pumps, enzymatic inactivation of the antimicrobial compound, and point mutations in the 50S ribosomal subunit [91,93]. In *Campylobacter*, resistance is attributable to mutations in the 23S rRNA gene that modifies the drug binding site, alterations to the protein composition of the target site, and enzymatic methylation of the macrolide molecule [11,45,74,86,93,94,95,96]. The most observed resistance mechanisms in *Campylobacter* are point mutations at positions 2074 and 2075 in the V domain of the 23S rRNA gene [11,25,45,74]. Three copies of this gene exist in *Campylobacter*, and resistance mutations are often found on all three copies in resistant isolates [97,98]. A2075G is the most common resistance-associated mutation, while other mutations, including A2074G and A2074C, are associated with considerably decreased macrolide susceptibility [25,45,99,100,101] (Table 1). Some strains of *C. jejuni* and *C. coli* pose the *erm(B)* gene either within the chromosome or via a plasmid, which encodes for the rRNA methylating protein Erm(B) [25,95,96]. The *erm(B)* gene is sufficient to provide considerable macrolide resistance [95]. The multidrug efflux system *cme*ABC is associated with macrolide resistance, acting in synergism with target mutations and ribosomal protein mutations L4 and L22, providing effective resistance [25,99].

### 3.5. Resistance to Aminoglycosides

Aminoglycosides act to inhibit bacterial protein synthesis via binding to the 30S ribosomal subunit, specifically the decoding site of the A site, resulting in alterations to protein expression and interference in the tRNA translocation between the A-site and P-site [102,103]. Aminoglycoside resistance is mediated via one of five mechanisms: efflux pumps removing the antimicrobial compound from the intracellular matrix; inhibition of drug binding via mutations or 16S RNA methylation at the drug binding sites; enzymatic deactivation of the antimicrobial molecule via modifications of -NH2 and -OH groups in the sugar moieties and 2-deoxystreptamine nucleus; and active swarming, a non-specific adaptive resistance mechanism observed in *P. aeruginosa* [102,104,105,106].

*Campylobacter* spp. poses several aminoglycoside-modifying enzymes such as aminoglycoside phosphotransferase (types I, III, IV, and VII), aminoglycoside acetyl transferase, aminoglycoside adenylotransferase, and aminoglycoside nucleotidyltransferase [16,107,108] (Table 1), with enzymatic modification being the most prevalent resistance mechanism present in *Campylobacter*. These enzymes fall into one of three categories based on their site of action and substrates: aminoglycoside phosphotransferases, aminoglycoside acetyltransferases, and aminoglycoside adenyltransferases [22,108]. All classes act similarly, acting to alter the aminoglycoside molecule to reduce its attraction to the A-site of the bacterial rRNA [22,103]. In *Campylobacter,* aminoglycoside phosphotransferase is the most common aminoglycoside-deactivating enzyme, acting to phosphorylate the 3’hydroxyl group of the aminoglycoside molecule [107]. Alpha-7, a gene encoding for a kanamycin phosphotransferase, has been found within *C. jejuni* on a plasmid that also carries tetracycline resistance genes [109]. While sharing a 55% sequence similarity to alpha-3, a resistance gene in streptococci, a G-C ratio of 32.8% indicates that the gene may be specific to *Campylobacter* [109]. The presence of alpha-3 genes in both *Campylobacter* and gram-positive organisms provides evidence of resistance gene transfer between gram-positives and gram-negatives [36].

## 4. Factors Influencing Antimicrobial Resistance in *Campylobacter*

### 4.1. Irrational Use of Antibiotics and the Role of the Environment

The overuse and misuse of antibiotics in veterinary and human medicine is one of the main causes of AMR in *Campylobacter* [110]. The emergence of resistance in humans is facilitated by the inappropriate prescribing of antibiotics [111]. Similarly, antibiotics are frequently employed as growth boosters or as preventative measures in animal agriculture, exposing *Campylobacter* to sub-lethal antibiotic concentrations that favour resistant strains [14]. The widespread use of fluoroquinolones, such as ciprofloxacin, in poultry farming has been strongly linked to the emergence of fluoroquinolone-resistant *Campylobacter* strains, which complicates the treatment of human infections [22]. Fluoroquinolone resistance in *Campylobacter* can be transferred to consumers who handle raw meat or consume undercooked meat [112].

Environmental factors, such as contamination of soil, food, and water with antibiotic residues or resistant bacteria, also play a role in shaping AMR in *Campylobacter* [113]. *Campylobacter* spp. is resilient and can survive in environmental matrices like water and soil, spreading resistance genes beyond farm environments [114]. Resistance can spread across the environment through manure contaminated with resistant *Campylobacter* or agricultural runoff containing antibiotics [14]. Wildlife can also act as carriers of resistant strains, spreading them across different ecosystems. The persistence of *Campylobacter* in biofilms, which protect bacteria from environmental stress and antimicrobial agents, further exacerbates the problem, as biofilms can serve as reservoirs for resistant strains [115].

### 4.2. Genetic Adaptability of the Pathogen

The genetic adaptability of *Campylobacter* significantly contributes to its ability to develop and disseminate resistance [115]. This adaptability is largely driven by its high genomic plasticity, which enables the rapid acquisition and incorporation of resistance genes from other bacteria in the environment [17]. The pathogen exhibits high genomic plasticity, allowing it to acquire resistance genes through horizontal gene transfer mechanisms, such as conjugation, transformation, and transduction [116]. These mechanisms allow *Campylobacter* to exchange genetic material with other members of the gut microbiota in both animals and humans, significantly enhancing its resistance capabilities [16]. *Campylobacter* can rapidly incorporate resistance genes from other bacteria in the gut microbiota of animals or humans, making it highly adaptable and selective to pressure [117]. Furthermore, resistance can be conferred and persistently transmitted via point mutations in genes that antibiotics target, such as the *gyr*A gene for fluoroquinolones. These mutations not only provide a survival advantage under selective pressure but are also stably inherited, ensuring resistance persists in subsequent generations [118].

The relatively small genome of *Campylobacter* and lack of robust DNA repair mechanisms further enhance its evolutionary flexibility [119]. It exhibits a natural competency for DNA uptake from its surroundings, allowing it to incorporate exogenous genetic material during stress or exposure to antimicrobials. These adaptations enable *Campylobacter* to thrive under selective pressures, including antibiotic treatments and environmental challenges [17]. Additionally, the ability of *Campylobacter* to form biofilms provides a protective niche for bacterial populations, facilitating the survival and exchange of resistance genes within these communities [120].

Beyond genetic factors, consumer awareness and behaviour indirectly influence the spread of resistant *Campylobacter*. Poor food handling practices, cross-contamination, and undercooking of poultry in kitchens can contribute to the spread of resistant *Campylobacter*. Also, inadequate public awareness regarding the risks associated with AMR and the importance of using antibiotics responsibly can perpetuate misuse. For instance, misuse of antibiotics in treating minor infections or failure to complete prescribed courses contributes to selective pressures that drive resistance. Public health campaigns emphasize proper food safety practices and responsible antibiotic usage, and the implications of AMR could play a vital role in mitigating the spread of resistant *Campylobacter* [121].

## 5. Intervention Strategies Used to Control *Campylobacter*

### 5.1. Pre-Harvest and Post-Harvest Intervention Strategies

**Figure 2:**
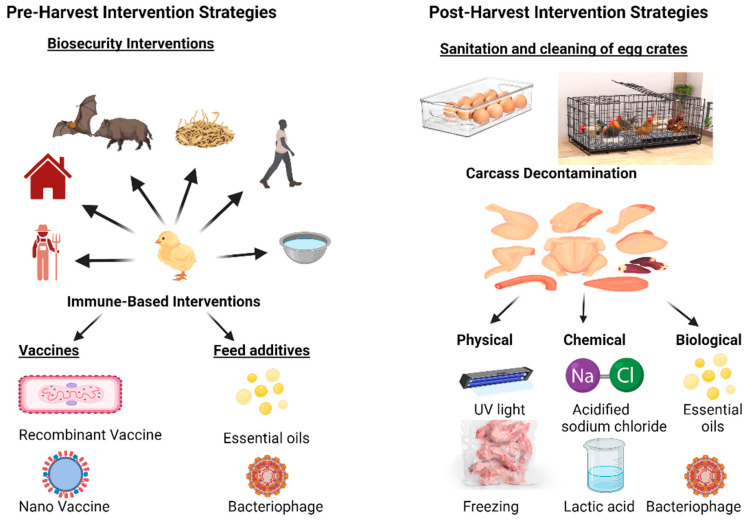
Intervention strategies used to tackle *Campylobacter*.

Controlling *Campylobacter*, one of the main causes of bacterial gastroenteritis, is important for public health [15]. Chickens are the primary source of infection in humans, with 50–80% of recorded cases of campylobacteriosis in Europe linked to ingesting poultry products contaminated with this bacterium [122]. To lessen the burden of these bacteria in chickens, intervention measures have been put in place, which include pre-harvest and post-harvest intervention strategies [4] (Figure 2).

#### 5.1.1. Pre-Harvest Intervention Strategies

Pre-harvest strategies aim to reduce *Campylobacter* colonization at the farm level, preventing the bacteria from reaching slaughterhouses. Strict biosecurity protocols form the cornerstone of pre-harvest strategies. Measures such as limiting farm access, establishing single-species poultry houses, pest control, and maintaining sanitary living conditions significantly reduce pathogen introduction and spread [123]. Immune-based strategies include vaccine development targeting *Campylobacter* colonization in poultry, which is a promising area of research. While commercial vaccines are not yet widely available, ongoing studies are exploring effective candidates that inhibit bacterial colonization. Bacterin-based vaccines and subunit vaccines targeting bacterial surface proteins show potential [4,124]. Incorporating feed additives such as probiotics, prebiotics, essential oils, organic acids, and the use of bacteriophages has shown promise in reducing *Campylobacter* loads in poultry intestines. Probiotics, such as *Lactobacillus* strains, outcompete *Campylobacter* and produce antimicrobial compounds [125]. Prebiotics, like fructooligosaccharides, enhance the growth of beneficial gut microbiota, indirectly inhibiting *Campylobacter* [126]. Bacteriophages, which are viruses that target specific bacteria, offer an eco-friendly and precise approach to *Campylobacter* reduction without affecting beneficial microbiota [127]. Essential oils, including thyme and oregano oils, disrupt bacterial membranes and exhibit potent antimicrobial effects [128]. Besides these, genetic and breeding approaches may also prove to be useful. Breeding chickens with innate resistance to *Campylobacter* colonization is an emerging avenue. Genetic studies have identified markers associated with reduced susceptibility, and selective breeding programs may help to develop resistant poultry lines [129].

While pre-harvest strategies are essential, they are not sufficient on their own to control *Campylobacter* in poultry. Post-harvest measures are vital in minimizing contamination during poultry slaughter, processing, and packaging of poultry products. These include cleaning and sanitizing slaughter plants, as well as disinfecting egg crates and eggshells. Carcass decontamination is another critical intervention to reduce the bacterial load. Regular disinfection of slaughterhouses and processing equipment helps to prevent bacterial cross-contamination. Additionally, using warm water combined with effective disinfectants is effective in removing residual *Campylobacter* from surfaces and equipment [130].

#### 5.1.2. Post-Harvest Intervention Strategies

Carcass decontamination, which falls under post-harvest measures, is another intervention strategy used to lower the bacterial presence on the surface of poultry meat before it reaches consumers. It is arguably the most impactful intervention strategy used to tackle *Campylobacter* in the sense that it is the most important stage in the food production process where *Campylobacter* contamination can be minimized effectively before it reaches consumers [131]. Unlike pre-harvest strategies, carcass decontamination addresses contamination that occurs during the processing of poultry. Since cross-contamination can happen at different stages in the slaughterhouse, effective decontamination methods are essential to ensure the meat is safe for consumption. This strategy, which uses physical, chemical, or biological methods, can be fully implemented with the introduction of mandatory government food safety regulations at the later stage of the food chain [4].

By using physical methods, lactic acid, trisodium phosphate, or acidified sodium chlorite can be applied to poultry carcasses to reduce the pH on the surface of poultry meat [132]. Additionally, poultry meat can be frozen at lower temperatures to significantly reduce *Campylobacter* levels and kill bacterial cells [4].

As consumer demand for safe, high-quality, and chemical-free poultry products increases, natural biological interventions for decontaminating *Campylobacter* are gaining prominence [128]. Essential oils disrupt bacterial membranes, offering strong antimicrobial effects. Bacteriophages provide a precise, eco-friendly technique to target and kill *C. jejuni* without affecting beneficial microbes [127]. Similarly, bacteriocins such as nisin inhibit bacterial growth by damaging cell walls, while probiotics like *Lactobacillus* outcompete pathogens and produce antimicrobial compounds [4]. These strategies not only enhance food safety, but also meet consumer preferences for sustainable and minimally processed food solutions.

## 6. Discussion and Conclusions

Understanding the parameters involved in antimicrobial resistance (AMR) in *Campylobacter* is crucial for developing sustainable intervention plans. This review highlights the growing public health significance of AMR in *Campylobacter* and emphasizes the urgent need to safeguard the effectiveness of fluoroquinolones and macrolides—key antibiotics in the treatment of campylobacteriosis. Alarmingly, resistance to these first-line treatments is rising, driven by misuse and overuse in both human medicine and the poultry industry. This necessitates stringent biosecurity measures, increasing production costs, while outbreaks linked to contaminated poultry can result in expensive recalls and trade restrictions. Additionally, subclinical infections in poultry can also lead to reduced weight gain and poor feed efficiency, impacting productivity. Overall, *Campylobacter* contamination not only threatens consumer safety but also imposes substantial economic and reputational costs on the poultry industry. For example, fluoroquinolone resistance, driven primarily by *gyr*A gene mutations, has reached critical levels globally, compromising the effectiveness of these drugs. Similarly, while resistance to macrolides remains less common, its emergence in certain regions is concerning, given that macrolides are frequently the first-line treatment for *Campylobacter* infections. These trends underscore the urgent need for a multifaceted approach to combat AMR in *Campylobacter*.

Addressing the escalating threat of AMR in *Campylobacter* requires coordinated global action, as it affects both developed and developing countries. Effective interventions must involve all stakeholders to ensure a unified response. Continuous global surveillance efforts are essential to monitor AMR trends and detect emerging resistance patterns in *Campylobacter*. Incorporating whole-genome sequencing (WGS) into these surveillance efforts offers deeper insights into resistance mechanisms and epidemiological relationships [133].

The misuse and overuse of antibiotics in human healthcare and poultry industry must be addressed. Policies aimed at limiting the use of antibiotics critical for human health in food-producing animals need to be strengthened and rigorously enforced. Alternative therapeutic strategies, including probiotics, antimicrobial peptides, and bacteriophages, have shown potential as effective non-antibiotic treatments for *Campylobacter* infections and should be actively pursued and promoted [134].

Both developed and developing nations have a pivotal role in combating AMR by enhancing regulations and implementing mechanisms to reduce the risk of *Campylobacter* infections and curb antibiotic use. This can be achieved by educating healthcare professionals and the public about proper antimicrobial use, making sure that practices like not washing raw chicken are implemented with clear reasons provided on how aerosols or spills generated can contaminate surfaces and lead to infections, particularly in the immunocompromised, as well as training food handlers in safe food handling, proper cooking, and improving hygiene practices. Additionally, investing in healthcare infrastructure to expand laboratory capacity for accurate diagnosis and susceptibility testing, along with improving access to clean water, sanitation, and hygiene facilities, can help to reduce infection transmission [135]. It is alarming that *Campylobacter* remains a neglected pathogen in African countries because of poor diagnosis and the barriers in culturing it due to its microaerophilic nature. It has often been seen as a carrier organism in young children in resource-limited countries, where it can contribute to stunting and poor cognitive development [3].

Adopting a One Health approach, which integrates efforts across human, animal, and environmental health sectors, is vital for addressing the interconnected challenges of AMR in *Campylobacter* [136]. This includes implementing integrated surveillance systems across sectors to monitor and manage resistance comprehensively. Supporting research and development is equally critical, with a particular focus on developing new antimicrobials, alternative therapies, and rapid diagnostic tools. Tackling underlying socioeconomic issues, such as poverty and limited access to healthcare, is also essential to reducing antibiotic misuse. Furthermore, enforcing stricter regulations on antimicrobial use in both human medicine and animal production can strengthen stewardship and help mitigate antimicrobial resistance. By integrating these strategies, the global community can address the rising threat of AMR in *Campylobacter* and preserve the efficacy of antibiotics for future generations.

## Figures and Tables

**Table 1:** Resistance genes and mutations conferring resistance to various antimicrobial classes in *Campylobacter*.

Genes or Mutation Conferring Resistance	Class of Antibiotic, Antibiotic	References
*bla*_OXA-61-like_, *bla*_OXA-184-like_, *bla*_OXA-576-like_ G57T promoter mutation upstream of the *bla*_OXA-61-like_ A69 deletion in the promoter upstream of the *bla*_OXA-61-like_	Beta-lactams, penicillin	[40,43,44]
23S rRNA gene mutation A2074→C, A2074→G, A2075→G	Macrolides, erythromycin	[21,45,46,47,48,49]
*tet*(O)-like *tet*(O/M/O) mosaic gene *tet*(O/32/O) mosaic gene	Tetracycline, tetracycline	[50,51]
*gyr*A gene mutations leading to the following changes: D90N, A70T, T86K, P104S and T86I	Fluoroquinolones, ciprofloxacin	[36,52,53,54]
*aac(3)*, *aac(6’)-Ib* (3-N-aminoglycoside acetyltransferase genes) *aph(2″)-Ig*, *aph(2″)-If* (aminoglycoside phosphotransferase) *sat-4* (streptothricin acetyltransferase) *ant6*, *ant2*, *antA*, *antB* (adenylyltransferase)	Aminoglycosides, gentamicin, amikacin, kanamycin, netilmicin, spectinomycin	[55,56,57,58,59,60,61,62]
*lnu*(AN2) (Lincosamide nucleotidyltransferase)	Lincosamides, clindamycin	[44]
*cat* (chloramphenicol acetyltransferase)	Chloramphenicol, chloramphenicol	[44,63]

## Data Availability

No new data were created in this study. Data sharing is not applicable to this article.
